# Supplementary material for: Examination of the Functional Relationship between PD-L1 DNA Methylation and mRNA Expression in Non-Small-Cell Lung Cancer
Source: Cancers (Basel). 2023 Mar 22;15(6):1909. doi: 10.3390/cancers15061909 (PMC10047551; doi:10.3390/cancers15061909)
Supplement: Supplementary file 1 [file cancers-15-01909-s001.zip › cancers-2234133-supplementary.pdf]

Supplemental file.

Table of content:

P2-P4: Supplemental material and

methods P5: Supplemental figure S1

P6: Supplemental figure S2

P7: Supplemental figure S3

P8: Supplemental figure S4

P9: Supplemental figure S5

P10: Supplemental figure S6

P11: Supplemental figure S7

P12: Supplemental table S1

P13: Supplemental table S2

Supplemental material and methods

DNA and RNA extraction

DNA from NSCLC cell lines was extracted using MasterPure DNA purification kit (Epicentre, Madison, WI, USA, MC85200) and from HEK293T cells using E.Z.N.A. Tissue DNA Kit (Omega Bio-Tek, Norcross, GA, USA, D3396) followed by treatment with RNase A (Omega Bio-Tek, AC118). DNA from five sections of 10 μm thick formalin-fixed paraffin-embedded tumor tissue was extracted using xylene and QIAamp DNA FFPE Tissue Kit (Qiagen, Venlo, Netherlands, 56404). DNA concentration and purity were measured by a NanodropTM spectrophotometer (Thermo Scientific). RNA from NSCLC cell lines used for correlation analyses between *PD-L1* mRNA expression and *PD-L1* methylation in NSCLC cell lines was extracted using an RNeasy Mini kit on a QIAcube instrument (Qiagen). RNA from IFN-γ stimulated cells and Cas9 sgRNA transduced cells was extracted using Trizol (Sigma-Aldrich, Saint Louis, MO, USA, T9424). Total RNA from five sections of 10 μm thick formalin-fixed paraffin-embedded tumor tissue was extracted using xylene and miRNeasy FFPE kit (Qiagen, 217504). The concentration and purity of RNA were measured with a NanodropTM spectrophotometer (Thermo Scientific, Waltham, MA, USA).

RNA expression analyses

cDNA synthesis on RNA from NSCLC cell lines was performed with an iScript cDNA synthesis kit (Bio-Rad, Hercules, CA, USA, 170-8890). First-strand cDNA synthesis on RNA from FFPE tumor tissue was performed on 1 ug RNA with SuperScript II Reverse Transcriptase kit (Invitrogen, Carlsbad, CA, USA, 18064) using 2 ϱmol of each of the following reverse primers; *TBP* 5′-CCCAACTTCTGTACAACTCTAGC-3′, *PD-L1* 5′-GGTGACTGGATCCACAACCA-3′ and *PD-L2* 5′-ACAGGTCTTTTTGTTGTGTCTTTTG-3′.

RT-qPCR was performed using LightCycler 480 platform (Roche, Basel, Switzerland) with the following settings: Pre-incubation at 95 °C for 15 min, 40 cycles of PCR amplification (95 °C for 30 sec, primer-specific annealing temperature for 30 sec, 72 °C for 30 sec) and final elongation at 72 °C for 1 min. Each reaction contained 1 μL cDNA (10 ng/μL), 0.125 μL forward primer (10 ϱmol/μL), 0.125 μL reverse primer (10 ϱmol/μL), 5 μL RealQ Plus 2x Master Mix Green (Ampliqon, Odense, Denmark, A323402) and 3.750 μL Nuclease-free H2O. Analysis of data was done using the X_0 method with *TBP* or *ACTB* mRNA expression as a reference gene [1]. Primer efficiencies were determined from a standard curve. For correlation analysis between *PD-L1* mRNA expression and *PD-L1* methylation in NSCLC cell lines the following primers and annealing temperature was used; *PD-L1* forward 5′-CCCCATACAACAAAATCAACCA-3′ and reverse 5′-TGCTTGTCCAGATGACTTCG-3′ (60 °C), and *ACTB* forward 5′-GGCGGCACCACCATGTACCCT-3′ and reverse 5′-AGGGGCCGGACTCGTCATACT-3′ (60 °C). For mRNA expression analysis of Cas9 sgRNA transduced cells, IFN-γ stimulated cells, and tumor tissue the following primers and annealing temperature were used; *PD-L1* forward 5′-ACTGTGAAAGTCAATGCCCCA-3′ and reverse 5′-GGTGACTGGATCCACAACCA-3′ (60 °C), and *TBP* forward 5′-AGGAGCCAAGAGTGAAGAACAG-3′ and reverse 5′-CCCAACTTCTGTACAACTCTAGC-3′ (60 °C).

DNA methylation analyses

For bisulfite pyrosequencing DNA was processed with an EpiTect Bisulfite kit (Qiagen, 59104). For pyrosequencing genomic regions were amplified by PCR using a PyroMark PCR kit (Qiagen, 978703) with the program: 95 °C for 15 min, 45 cycles of (94 °C for 30 sec, assay-specific annealing temperature for 30 sec, 72 °C for 1 min) and 72 °C for 10 min. The PCR reaction included 0.5 µL bisulfite-treated DNA from NSCLC cell lines (approximately 6-12 ng), 6 uL from HEK293T cells (approximately 25-75 ng), and 6 uL from tumor tissue (approximately 25 ng), 0.5 µL forward and reverse primer (10 ϱmol/µL), 12.5 µL PyroMark PCR Master Mix, 2.5 uL Coral Load, 2 uL $MgCl_2$ (25 mM) and Nuclease-free H2O to a total volume of 25 µL. For cg19724470 and cg(chr9:5,450,951) (annealing temp. 56°C) was used primers 5′- GGTAGTATAGGATAGGTAGTTATTT-3′ and 5′-Biotin-TTAAATACTACAACTAAAATAACTTACACAAA-3′. For cg04478497 and cg00975815 (annealing temp. 54 °C) was used primers 5′-GGTTATGTGTAGTTTGTATAGTTGTT-3′ and 5′-Biotin-AATAAATTTTCCCTCTTAATACCTCTC-3′. Biotin-labeled single-stranded amplicons were isolated from 20 µL PCR product using Qiagen PyroMark Q24 Vacuum Workstation and pyrosequenced using PyroMark Q24 reagents (Qiagen, 970802). For cg19724470 and cg(chr9:5450951) was used primer 5′-TGATGTTAGGTTGGAGGT-3′ and for sequencing of cg04478497 and cg00975815 primer 5′-GGAATATTAATTTTTTGTTTT-3′. PCR and pyrosequencing assay efficiencies were validated using standard 0% methylated DNA (EpiTect Control DNA, Qiagen, 59568), 100% methylated DNA (Universal Methylated Human DNA Standard from Zymo Research, Irvine, CA, USA, D5011), and 50% methylated DNA (a mix of 0% and 100% methylated DNA after bisulfite-treatment). All samples were PCR amplified and pyrosequencing was performed in at least duplicates. Data were analyzed with PyroMark Q24 software. For clonal bisulfite sequencing, PCR primers were designed to amplify the 3′ end of the CpG island and part of the associated shore region. PCR was performed with HotStarTaq DNA polymerase (Qiagen) using 2 µL bisulfite-converted DNA (approximately 15 ng), 2.5 µL 10 X PCR buffer, 0.5 µL dNTP, 0.15 µL HotStarTaq DNA polymerase, 10 pmol forward primer 5′-GTTGTTTTGGGTAGAGGTGG-3′ and reverse primer 5′-CCAACAAATAATTAACTCTACTACC-3′ in a total volume of 25 µL with the following settings: Heating at 95°C for 15 min, 45 cycles of PCR (94°C for 30 sec, 56°C for 30 sec, 72°C for 1 min) and final elongation at 72°C for 10 min. The 301 bp PCR product was cloned using TOPO® TA Cloning® Kit for Sequencing (Invitrogen, Life technologies, K4575J10). Positive clones were identified by PCR-based screening using M13 forward 5′-GTAAAACGACGGCCAGTG-3′ and reverse 5′-CAGGAAACAGCTATGACC-3′ primers. The purified PCR product was Sanger sequenced using T3 primer and sequences presenting reverse orientation of the insert were used for assessment of methylation.

Examination of CRISPR/Cas9-mediated deletions

The genomic region was amplified using 125 ng DNA and Phusion High-Fidelity PCR Master Mix with HF buffer (Thermo Scientific, F531L) with the following PCR program; Initial denaturation at 98 °C for 30 sec, 35 cycles of (98 °C for 10 sec, 58 °C for 30 sec, 72 °C for 20 sec) followed by final elongation at 72 °C for 10 min using *PD-L1* primers forward 5′-ACTTTAGGACGGAGGGTCTC-3′ and reverse 5′-GGCCAAGGTCAATGTGTCTA-3′. Sanger sequenced PCR products were analyzed with Synthego ICE software (https://labs.synthego.com/).

dSaCas9-TET1 and dSpCas9-DNMT3A fusions

For establishing cells with stable expression of dCas9 fusions HEK293T cells (150.000-300.000 cells/well) were seeded in 6-well plates. The next day cells were co-transfected with 200 ng pCMV-hyPBase [2],

1.8 μg PB-TRE-dSaCas9-TET1-Puro, PB-TRE-dSaCas9-dTET1-Puro, PB-TRE-dSpCas9-DNMT3A-Hygro or PB-TRE-dSpCas9-dDNMT3A-Hygro using 5 μL Lipofectamine™ 3000 Transfection Reagent and 4 μL P3000™ Reagent (Invitrogen™, L3000-008). Two days after transfection 0.5 μg/mL puromycin (Sigma-Aldrich, P8833) or 200 μg/mL hygromycin (Invitrogen, 10687010) was added and selection progressed for 10 days. Expression of dCas9-fusions was induced by 1 μg/mL Dox (Sigma-Aldrich, D9891). For protein expression analysis, cells were treated with Dox for 24h before cell harvest for western blot. For targeted DNA methylation editing cells were treated with Dox for 0.5h - 1h before lipofectamine transfection using 2 μg sgRNA vector/well and continued for 48h. The doxycycline-containing medium was replaced once per day.

Protein Purification and western blotting

Cells were harvested by trypsinization using 0.05% Trypsin-EDTA and washed twice in PBS. Cells were then dissolved in 100 μL lysis buffer (50 mM Tris HCl, 5 mM EDTA, 1 mM DTT, 10 μg/mL aprotinin, and 1 mg/mL Soybean Trypsin Inhibitor) with 0.5 % triton x-100 (Sigma-Aldrich, T8787) and incubated in three rounds of 3 min on dry ice and 3 min at 37°C. The samples were subsequently incubated for 30 min on ice. Protein was collected in the supernatant by centrifugation for 10 min at 10,000 rpm. Protein concentration was measured by the Bradford method.

2-40 μg protein was mixed with 5 μL Pierce™ Lane Marker Reducing Sample Buffer (Thermo Scientific, 39000) and diluted to a final volume of 17.5 μL with PBS. The samples were heated to 100°C for 10 min and loaded into a Mini-protean TGX 4-15% precast gel (Bio-Rad, 4561085). Proteins were separated in 1x Tris-Glycine-SDS buffer (Bio-Rad, 161-0732) at 40 mA for 45 min and blotted onto an Amersham™ Hybond® P Western blotting membrane (GE Healthcare, Chicago, IL, USA, GE10600069) in 1x Tris-Glycine buffer (Bio-Rad, 161-0734) at 60 V for 30 min on ice. The membrane was blocked in 5% bovine serum albumin (BSA) (Sigma-Aldrich, A2153) for 1h while shaking and incubated with primary antibody diluted in 5% BSA overnight at 4°C on a rotator. The primary antibodies used were anti-HA-tag 1:5000 (Cell signaling technology, 3724), anti-FLAG-tag 1:1000 (Sigma-Aldrich, F1804), and anti-H3 1:20,000 (Abcam, ab1791). The membrane was washed for 5 min three times in Western Blot (WB) wash buffer (1 mL Tween 20 (Sigma, *T9416*) diluted to a final volume of 1L in 1X Tris Buffered Saline (Fisher Scientific, BP2471)) and incubated with secondary antibody diluted 1:5000 in 5% BSA for 1h while shaking and protected from light. The secondary antibodies used were anti-Mouse Immunoglobulins/horseradish peroxidase (HRP) (Dako, Copenhagen, Denmark, P0447) and anti-Rabbit Immunoglobulins/HRP (Dako, P0448). The membrane was washed for 5 min five times in WB wash buffer. The membrane was developed with BM Chemiluminescence Western Blotting Substrate (POD) (Roche, 11500694001) and visualized in 10-sec exposure intervals using the ImageQuant™ LAS 4000 system (GE Healthcare).

References

1. Thomsen, R., et al., *Analysis of qPCR data by converting exponentially related Ct values into linearly related X0 values.* J Bioinform Comput Biol, 2010. **8**(5): p. 885-900.
2. Yusa, K., et al., *A hyperactive piggyBac transposase for mammalian applications.* Proc Natl Acad Sci U S A, 2011. **108**(4): p. 1531-6.

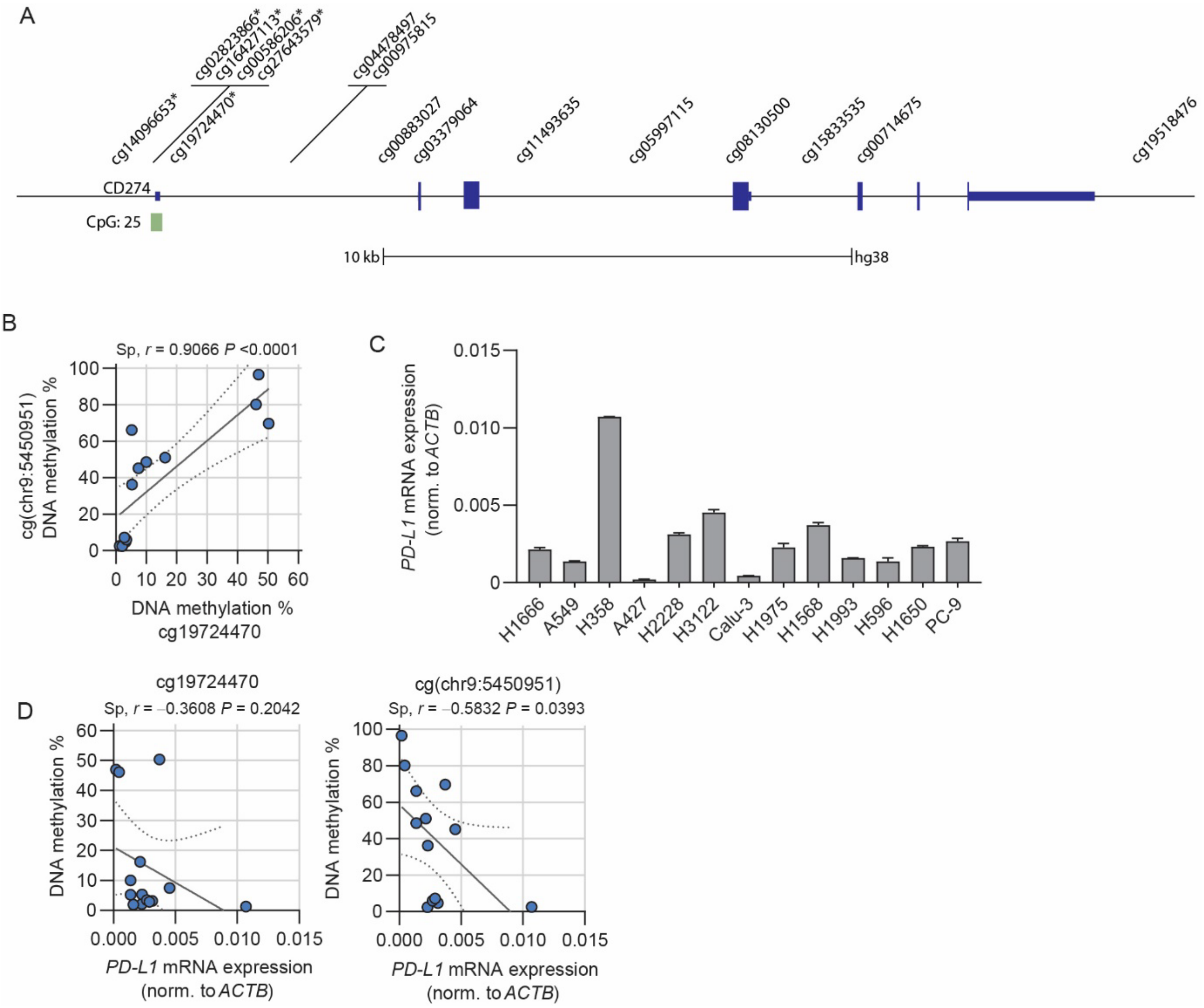

Figure S1: PD-L1 gene methylation and mRNA expression data (A) PD-L1 gene from UCSC Genome Browser (CRCh38/hg38). The merged PD-L1 consists of seven exons with the transcriptional start site located at the beginning of exon 2. The localization of CpG sites included in the 850K array that maps to PD-L1 is indicated. The CpG island (green) consists of 25 CpG sites. cg02823866, cg16427113, cg00586206, and cg27643579 are located within the CpG island and marked with asterisks. (B) Correlation analysis of DNA methylation. Spearman correlation coefficient and p-value are shown. Linear best fit (solid line) and 95% confidence intervals (dotted lines) are depicted. (C) *PD-L1* mRNA expression in NSCLC cell lines measured by RT-qPCR. *PD-L1* mRNA expression was normalized to ACTB. Mean ± SD of two RT-qPCR reactions. (D) Correlation analyses of *PD-L1* mRNA expression measured by RT-qPCR (cell lines shown in panel C and in addition HCC827 shown in Figure S2A) and DNA methylation of cg19724470 and cg(chr9:5450951). Spearman correlation coefficient (r) and p-value are given. Linear best fit (solid line) and 95% confidence intervals (dotted lines) are depicted on the plot.

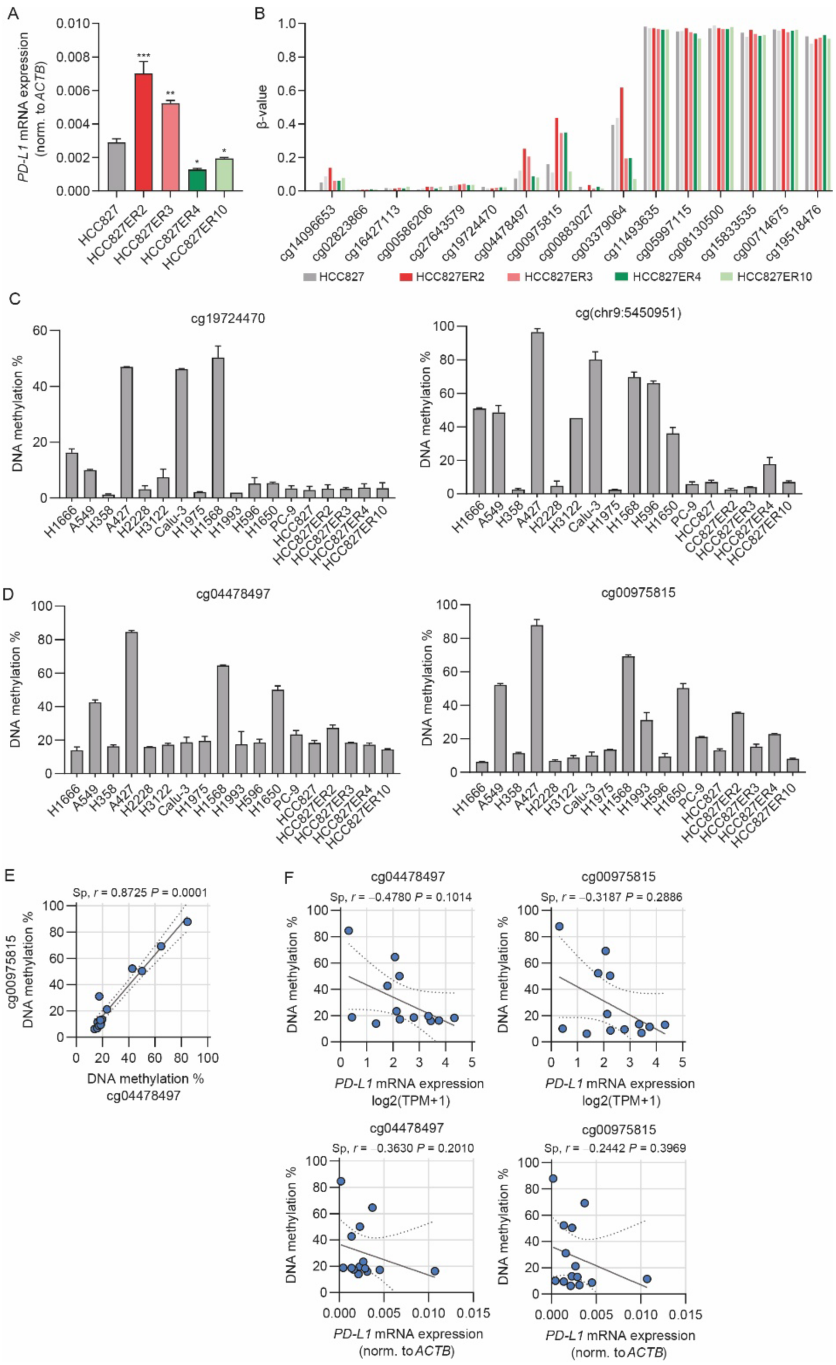

Figure S2. *PD-L1* methylation and mRNA expression in HCC827 cell clones. (A) *PD-L1* mRNA expression normalized to *ACTB* of HCC827 cells (grey) and HCC827ER clones with high (red) and low (green) *PD-L1* mRNA expression. Mean + SD. N = 2. Differences between HCC827 and HCC827ER clones were tested with Ordinary one-way ANOVA with correction for multiple comparisons using the Holm-Šidák method. Adjusted p-value * < 0.05; ** < 0.01; *** < 0.001. (B**)** β-values of CpG sites located 500 bp upstream and downstream of *PD-L1* were obtained from 850K array of HCC827 cells (grey) and HCC827ER clones with high (red) and low (green) *PD-L1* mRNA expression. (C) DNA methylation of cg19724470 and cg(chr9:5450951) in NSCLC cell lines including HCC827ER clones. Mean + SD of at least two pyrosequencing experiments. (D) DNA methylation of cg04478497 and cg00975815 in NSCLC cell lines including HCC827ER clones. Mean + SD of at least two pyrosequencing experiments. (E) Correlation analysis of DNA methylation. Spearman correlation coefficient and p-value are shown. Linear best fit (solid line) and 95% confidence intervals (dotted lines) are depicted. (F) Correlation analyses of *PD-L1* mRNA expression from the DepMap Portal (upper panels) or measured by RT-qPCR (Lower panels, cell lines shown in Figure S1C and in addition HCC827 shown in panel A) and DNA methylation of cg04478497 and cg00975815. Spearman correlation coefficient (r) and p-value are given. Linear best fit (solid line) and 95% confidence intervals (dotted lines) are depicted on the plot.

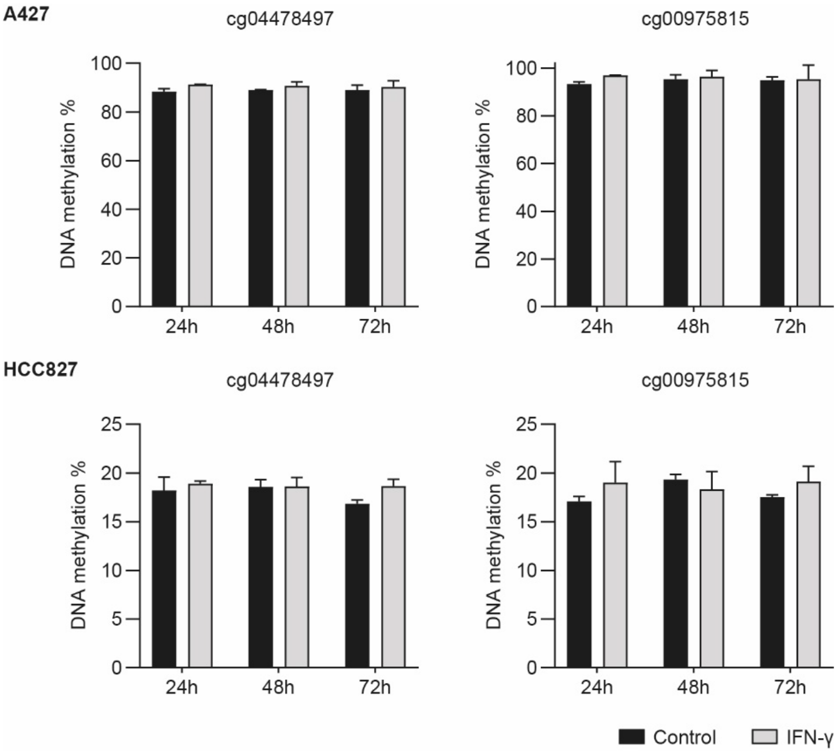

Figure S3: PD-L1 stimulation with IFNg. A427 cells (upper panel) and HCC827 cells (lower panel) were stimulated with 10 ng/mL IFN-γ for 24h, 48h, or 72h. DNA methylation of cg04478497 and cg00975815. Mean ± SD. N = 3 (except for A427 control and IFN-γ at 24h and HCC827 IFN-γ at 24h where N = 2). Significant differences between the control and IFN-γ treatment were tested with an unpaired t-test with correction for multiple comparisons using the Holm-Šidák method.

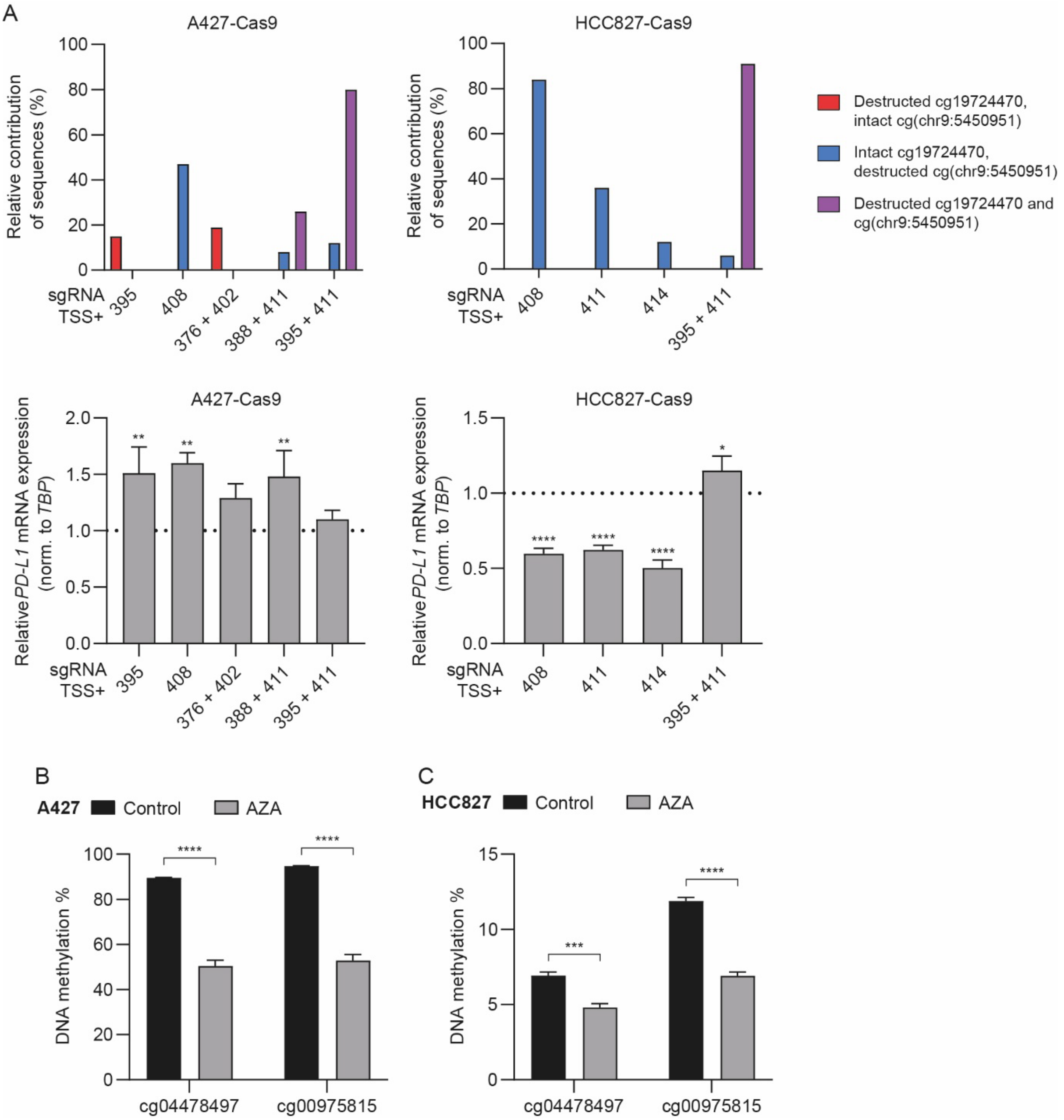

Figure S4 Characterization of PD-L1 regulation by DNA methylation (A) A427-Cas9 and HCC827-Cas9 cells were transduced with various single sgRNAs or in a combination of two sgRNAs together flanking the genomic region of *PD-L1* including cg19724470 and cg(chr9:5450951). Upper panel: After sequencing of transduced cells, each sample was screened for the following outcomes; the relative contribution of sequences with the destruction of cg19724470 while cg(chr9:5450951) were intact (red), cg19724470 were intact while cg(chr9:5450951) were destructed (blue) or if both cg19724470 and cg(chr9:5450951) were destructed (purple). Only transduced samples displaying a minimum of one of the described outcomes are depicted in the Figure. Lower panel: *PD-L1* mRNA expression normalized to *TBP* followed by normalization to cells transduced with control sgRNA (the dotted line represents normalized control sgRNA). Mean + SD of technical triplicates. One-way ANOVA with Holm-Šidák's multiple comparisons test was used to test for significance. Adjusted p-value * < 0.05; ** < 0.01; *** < 0.001; ****< 0.0001. (B-C) Effect of 48 h azacytidine (AZA) treatment for *PD-L1* methylation in (B) A427 cells and (C) HCC827 cells. Mean + SD of technical triplicates. Differences between the control and AZA0 were tested with an unpaired t-test with correction for multiple comparisons using the Holm-Šidák method.

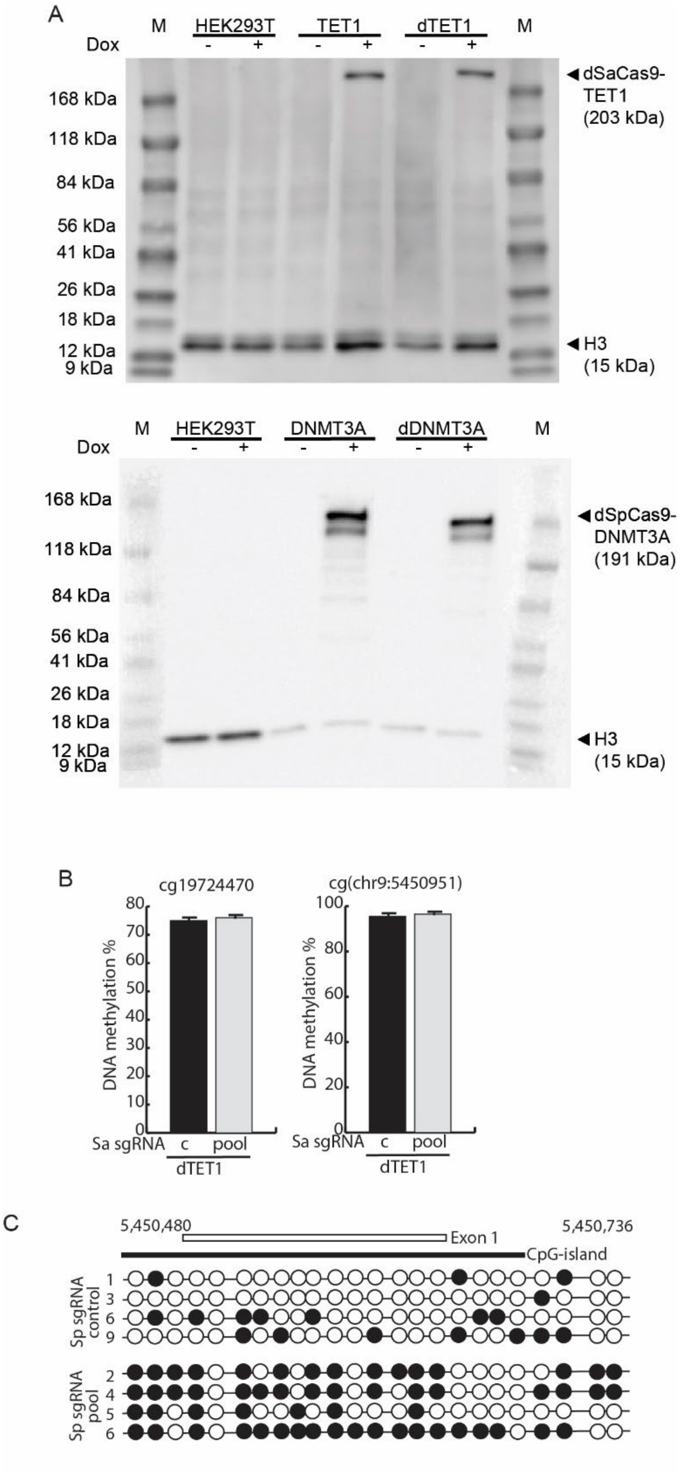

Figure S5 Additional dCAS methylation data (A) Validation of induced dSaCas9-TET1 and dSaCas9-dTET1 (203 kDa) expression (left panel) and dSpCas9-DNMT3A and dSpCas9-dDNMT3A (191 kDa) expression in HEK293T cells after 24 hours of stimulation with 1 μg/mL doxycycline by western blot. H3 was used as a loading control. (B) DNA methylation of cg19724470 and cg(chr9:5450950) in dSaCas9-dTET1 HEK293T cells transfected with Streptococcus aureus (Sa) control sgRNAor a pool of sgRNAs targeting the south shore of the *PD-L1* CpG island (pool). 48 hours after transfection the cells were analyzed for *PD-L1* methylation. (C) Determination of methylation of the 3′ region of the CpG-island and associated shore in HEK293T dSpCas9-DNMT3A cells transfected with Streptococcus pyogenes (Sp) control sgRNA or a pool of Sp sgRNAs targeting the north and south shores of the *PD-L1* CpG island. N = 4 sequences.

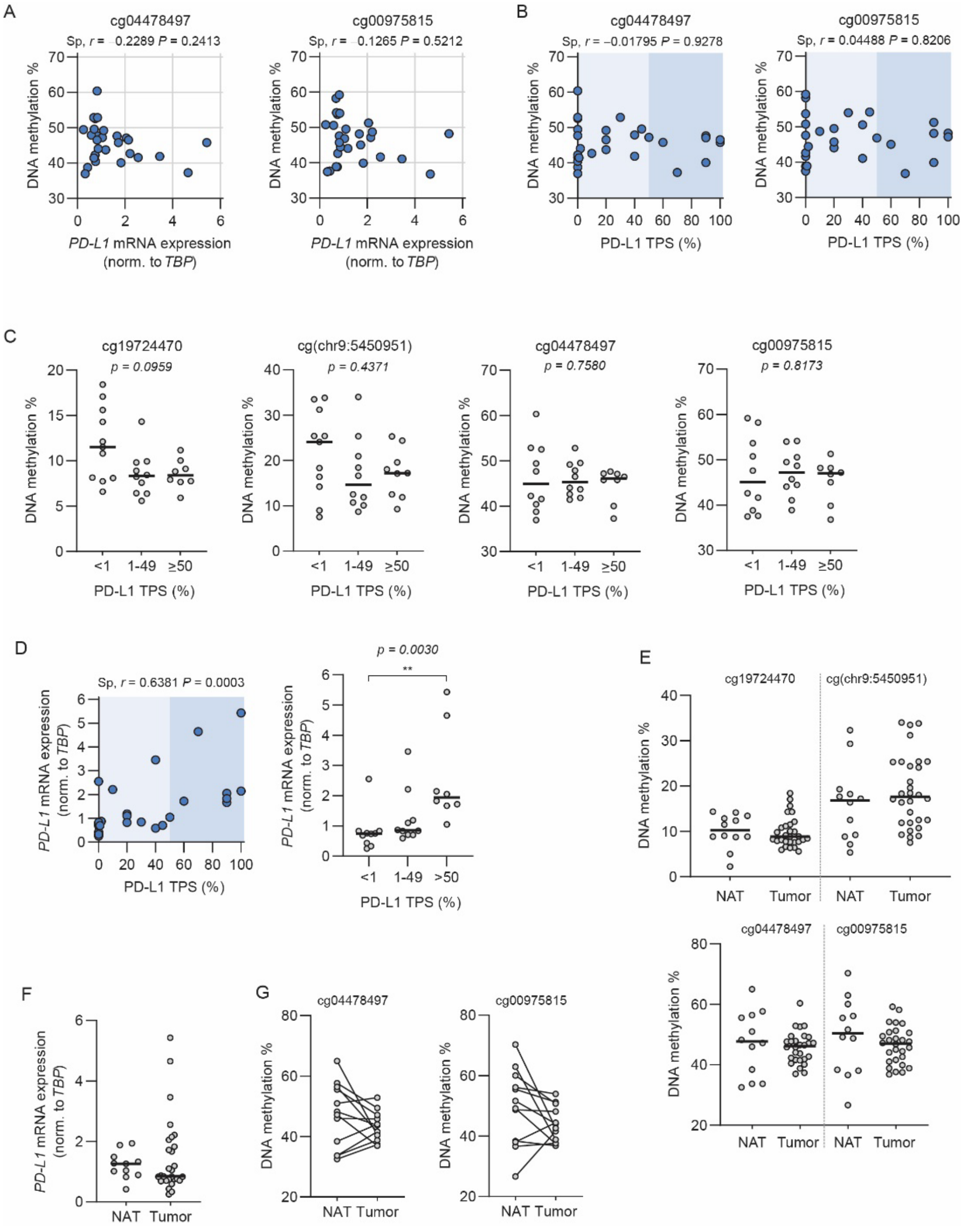

Figure S6 *PD-L1* methylation, *PD-L1* mRNA expression, and PD-L1 TPS (A) Correlation analysis of *PD-L1* mRNA expression and DNA methylation of cg04478497 and cg00975815, respectively, in NSCLC tumor tissue (N = 28). Spearman correlation coefficient (r) and the p-value are depicted above the graph. (B) Correlation analysis of PD-L1 TPS and DNA methylation of cg04478497 and cg00975815, respectively, in NSCLC tumor tissue (N = 28). Spearman correlation coefficient (r) and the p-value are depicted above the graph. Shading represents PD-L1 TPS subdivisions (<1%, 1-49%, and ≥50%). (C) DNA methylation of cg19724470, cg(chr9:5450951), cg04478497 and cg00975815 in tumor tissue from NSCLC patients subdivided according to PD-L1 TPS <1%, 1-49% and ≥50%. Kruskal-Wallis test with Dunn's multiple comparisons test was applied to test for the difference between medians of PD-L1 TPS <1%, 1-49%, and ≥50%. p-values are depicted. The line represents the median. (D) Similar to b and c but for *PD-L1* mRNA expression and TPS. (E) DNA methylation of cg19724470 and cg(chr9:5,450,951) and cg04478497 and cg00975815 in normal adjacent tissue (N = 12) and NSCLC tumor tissue (N = 29 for cg19724470, N = 30 for cg(chr9:5450951), and N = 28 for cg04478497 and cg00975815). Kruskal-Wallis test with Dunn's multiple comparisons test was applied to test for the differences between the median of normal adjacent tissue and tumor tissue. The line represents the median. (F) *PD-L1* mRNA expression normalized to *TBP* in normal adjacent tissue (N = 11) and NSCLC tumor tissue (N = 28). Mann-Whitney t-test was used to test for significance. (G) DNA methylation of cg04478497 and cg00975815 in normal adjacent tissue and tumor tissue connected by a line for each patient (N = 12). NAT; normal adjacent tissue. Adjusted p-value ** < 0.01.

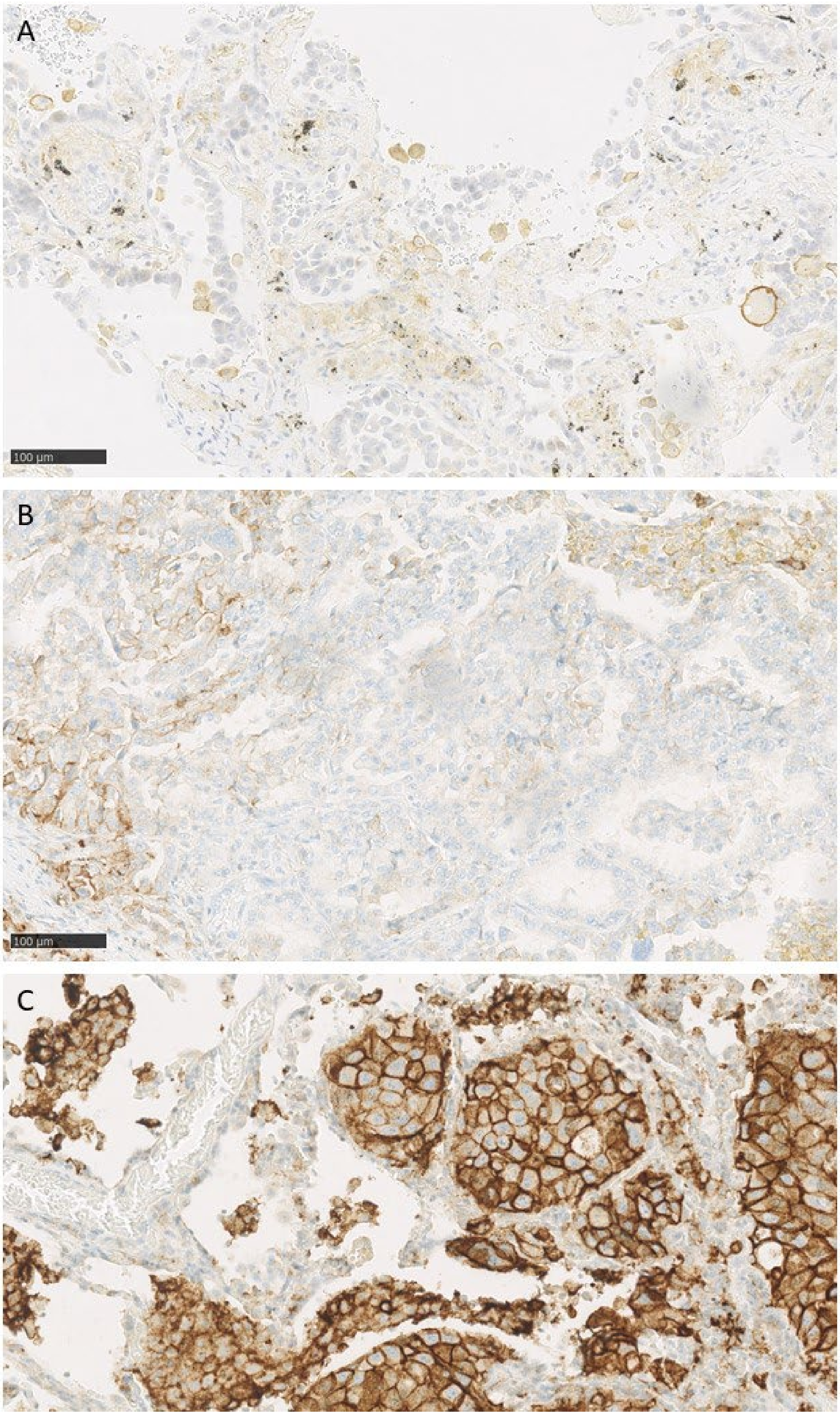

Figure S7 Images showing the three PD-L1 immunohistochemical staining patterns: <1% (A), 1-49% (B), >50% (C). The PD-L1 expression was reported as the proportion of neoplastic cells with complete or partly membranous staining, known as the tumor proportion score. Positive macrophages serve as internal positive controls.

Table S1 Details of NSCLC cell lines

NSCLC cell line	origin	charactristics	Mutation(s)
A549	ATCC, CRM-CCL-185.	Epithelial cell that was isolated from the lung of a 58-year-old White male with carcinoma.	KRAS, p.Gly12Ser.
H358	ATCC, CRL-5807. NCI-H358.	Epithelial-like cell that was isolated from the bronchiole of a male patient with bronchio alveolar carcinoma.	KRAS p.G12C. TP53 gene deletion. STK11, p.Gln37Ter.
H1666	ATCC, CRL-5885 NCI-H1666	Cell with epithelial morphology that was isolated from the lungs of a 50-year-old, White female patient with bronchoalveolar carcinoma.	BRAF p.G466V.
HCC827	ATCC, CRL-2868	Epithelial cell that was isolated from the lung of a White, 39-year-old female patient with adenocarcinoma.	EGFR p. delE746 - A750. TP53, p.Val218del.
H1650	ATCC, CRL-5883 NCI-H1650	Cell with epithelial morphology that was isolated from the lung tissue of a 27-year-old, male smoker with stage 3B, bronchoalveolar carcinoma.	EGFR p.delE746-A750. TP53, c.673-2A>G.
PC-9	PHE	pulmonary adenocarcinoma cell line.	EGFR p.delE746-A750. TP53, p.Arg248Gln.
H1993	ATCC, CRL-5909. NCI-H1993	Cell with epithelial morphology that was isolated from the lungs of a 47-year-old, White female patient with stage 3A non-small cell lung cancer: adenocarcinoma.	TP53, p.Cys242Trp.
H1568	ATCC, CRL-5876 NCI-H1568	Cell isolated from the lungs of a 48-year-old, White female patient with non-small cell lung cancer of type adenocarcinoma.	ATM, p.Gly506Phe. TP53, p.His179Arg.
H2228	ATCC, CRL-5935 NCI-H2228	Cell of type lung adenocarcinoma derived from a female nonsmoker with non-small cell lung cancer.	EML4-ALK fusion. ALK-PTPN3 fusion. TP53, p.Gln331Ter.
H1975	ATCC, CRL-5908. NCI-H1975.	Cell with epithelial morphology from the lungs of a nonsmoking female with non-small cell lung cancer.	EGFR, p.L858R, T790M. TP53, p.Arg273His. PIK3CA, p.Gly118Asp.
Calu-3	ATCC, HTB-55.	Epithelial cell isolated from lung tissue derived from a 25-year-old, White, male patient with lung adenocarcinoma who received prior therapy with cytoxan, bleomycin, and Adriamycin.	TP53, p.Met237Ile.
H596	ATCC, HTB-178. NCI-H596.	Epithelial-like cell that was isolated from the lung of a 73-year-old, White, male with lung adeno squamous Carcinoma.	TP53, p.Gly245Cys. RB1, p.Ser182fs*3. PIK3CA, p.Glu545Lys.
A427	ATCC, HTB-53.	Cell from lung adenocarcinoma (NSCLC) from male 52 years.	KRAS, p.Gly12Asp. CTNNB1, p.Thr41Ala.
H3122	See footnote* NCI-H3122.	Adenocarcinoma cell from the lung characterized by the presence of malignant glandular epithelial cells.	EML4-ALK fusion
HCC827ER2	Jakobsen et al. [1]	Erlotinib resistant cell subclone of HCCC827 possessing *MET*-amplification.	EGFR p.delE746-A750. TP53, p.Val218del. *MET*-amplification
HCC827ER3	Jakobsen et al. [1]	Erlotinib resistent cell subclone of HCCC827 possessing *MET*-amplification.	EGFR p.delE746 - A750. TP53, p.Val218del. *MET*-amplification
HCC827ER4	Jakobsen et al. [1]	Erlotinib resistent cell subclone of HCCC827 possessing epithelial-mesenchymal transition.	EGFR p.delE746 - A750. TP53, p.Val218del.
HCC827ER10	Jakobsen et al. [1]	Erlotinib resistent cell subclone of HCCC827 possessing epithelial-mesenchymal transition.	EGFR p.delE746 - A750. TP53, p.Val218del.

Mutation data were extracted from Cellosaurus (https://www.cellosaurus.org/). ATCC (American Type Culture Collection). PHE (Public Health England culture collections, Salisbury, UK). * H3122 was a generous gift from Adi Gazdar at UT Southwestern Medical Center, Dallas, USA.

1. Jakobsen, K.R., et al., *MET amplification and epithelial-to-mesenchymal transition exist as parallel resistance mechanisms in erlotinib-resistant, EGFR-mutated, NSCLC HCC827 cells.* Oncogenesis, 2017. **6**(4): p. e307.

Table S2 sgRNA sequences.

name	Cas9 variant	Sequence (5' – 3')	PAM (5' – 3')	Genomic strand	position
**Vector pLenti-sgRNA(SpCas9)-eGFP-Puro**					
control	SpCas9	ACGGAGGCTAAGCGTCGCAA			
376	SpCas9	CAGAATATAGCTCTGATGCT	AGG	+	+376
388	SpCas9	CTGATGCTAGGCTGGAGGTC	TGG	+	+388
395	SpCas9	TAGGCTGGAGGTCTGGACAC	GGG	+	+395
402	SpCas9	CAAGCAGCTGGCGGTGGACT	TGG	-	+402
408	SpCas9	TACTAGCAAGCAGCTGGCGG	TGG	-	+408
411	SpCas9	TGTTACTAGCAAGCAGCTGG	CGG	-	+411
414	SpCas9	TCATGTTACTAGCAAGCAGC	TGG	-	+414
**Vector pLenti-sgRNA(SaCas9)-eGFP-Puro**					
control	dSaCas9	ACGGAGGCTAAGCGTCGCAA			
U1	dSaCas9	CAGTAGAGCCAATTACCTGT	TGGGGT	+	+256
U2	dSaCas9	TTCTGAACACACAGTCGCAT	AAGAAT	-	+380
D1	dSaCas9	ACTTACTTAGATGCTGCAGC	TGGGAT	-	+491
D2	dSaCas9	GCAGGTCCAGGATCCCTGAA	CGGAAT	+	+546
D3	dSaCas9	ACCCAAAGGAGTCCTAAAAG	AGGAAT	+	+596
**Vector pLenti-sgRNA(SpCas9)-eGFP-Puro**					
control	dSpCas9	ACGGAGGCTAAGCGTCGCAA			
U1	dSpCas9	CAGTAGAGCCAATTACCTGT	TGG	+	+256
D1	dSpCas9	ACTTACTTAGATGCTGCAGC	TGG	-	+491
N1_Sp	dSpCas9	ACACCATCGTCTGTCATCTT	GGG	+	-741
N2_Sp	dSpCas9	CCTGATATTCTGCCACCCTA	AGG	-	-662

List of short guide RNA (sgRNA). Positions are given relative to the distance between the PAM-sequence and the transcriptional start site for *PD-L1*. Enzymatic inactivated Cas9 (dCas9). pyromycin resistence gene (Puro). Enhanced green fluorescens protein (eGFP). Staphylococcus aureus (Sa). Streptococcus pyogenes (Sp).
